# Factors That Influence Susceptibility Vessel Sign in Patients With Acute Stroke Referred for Mechanical Thrombectomy

**DOI:** 10.3389/fneur.2022.893060

**Published:** 2022-05-11

**Authors:** Manon Dillmann, Louise Bonnet, Fabrice Vuillier, Thierry Moulin, Alessandra Biondi, Guillaume Charbonnier

**Affiliations:** ^1^Neurology Department, University Hospital Centre Besancon, Besançon, France; ^2^Laboratoire de Recherches Intégratives en Neurosciences et Psychologie Cognitive - UR 481, Université de Franche-Comte UFR Sciences Médicales et Pharmaceutiques, Besançon, France; ^3^Interventional Neuroradiology Department, University Hospital Centre Besancon, Besançon, France

**Keywords:** stroke, ischemic stroke, susceptibility vessel sign, mechanical thrombectomy, magnetic resonance imaging, thrombus

## Abstract

**Background and Purpose:**

The presence of a Susceptibility Vessel Sign (SVS) in the acute phase of proximal occlusion ischemic stroke indicates the presence of deoxyhemoglobin in the thrombus. Thrombi composition changes over time. The aim of this study was to investigate whether the absence of SVS is associated with a shorter symptom onset to imaging time.

**Methods:**

We retrospectively analyzed all patients referred for mechanical thrombectomy at Besançon University Hospital between 1 January 2015 and 31 December 2020 for whom readable T2^*^-weighted imaging was available. We compared patient characteristics according to the presence or absence of an SVS. We also studied the subgroup for whom the exact symptom onset time was known. We performed a univariate statistical analysis, then a multivariate analysis on the variables that were statistically significant in the univariate analysis.

**Results:**

Of the 389 patients included, 309 (79.4%) were SVS+. We found no significant relationship between SVS– and the time between symptom onset and imaging in the whole cohort. In the multivariate analysis, SVS– was associated with anticoagulant treatment (*p* < 0.01), and SVS+ with age (*p* = 0.023) and carotid terminus occlusion (*p* = 0.042). In the known symptom onset subgroup, SVS– was significantly associated with a shorter symptom onset -imaging time (*p* < 0.001), and this was confirmed in the multivariate analysis (*p* = 0.011; OR 0.911; 95% CI [0.844; 0.972]).

**Conclusion:**

In the acute phase of proximal occlusion ischemic stroke, absence of SVS was associated with a shorter symptom onset–imaging time for patients with a known symptom onset time.

## Introduction

Ischemic stroke due to intracranial arterial occlusion can be linked to the presence of a Susceptibility Vessel Sign (SVS) on gradient-recalled echo magnetic resonance imaging (MRI) sequences. It is a complex radiological sign that has been reported to be associated with various parameters, like cardioembolic etiology (1–4), clinical outcome (5–9), or success of recanalization therapy (1, 10).

The SVS is a blooming artifact at the site of the thrombus. It is related to the presence of deoxyhemoglobin in the thrombus, which itself is linked to the presence of red blood cells (11–14). The composition of the thrombus, which blocks the blood flow, varies over time (15, 16). The oxyhemoglobin within the thrombus breaks down into oxygen and deoxyhemoglobin. We can therefore hypothesize that the likelihood of the presence of an SVS is related to the age of the thrombus. The relation between symptom onset (SO) time and SVS in literature is controversial, with studies finding different results (6, 7, 10, 17–19). The aim of this study was to investigate whether the absence of SVS is associated with a shorter SO to imaging time.

## Methods

We conducted a retrospective, single-center study using data from the Franche-Comté Stroke registry (20–22). All patients had previously been included in a single-center cohort study and their data was included in the present study on the basis of non-opposition, in accordance with local legislation.

### Population

We analyzed the data from all patients with acute stroke referred for a mechanical thrombectomy procedure at Besançon University Hospital between 1st of January 2015 and 31st of December 2020. We excluded patients who had not undergone a T2^*^-weighted MRI sequence in the acute phase, or whose imaging contained artifacts that made the images prohibitively difficult to interpret. We also excluded any patients whose symptom onset time was not recorded in the patient record.

### Clinical Data

Demographic data had been collected prospectively by the stroke unit and was obtained from the patient records for our study. The available data included the patient's age, sex, and treatment with an antiplatelet drug, anticoagulant, or both at admission. Data concerning the acute phase of the stroke had been collected prospectively by the on-call neurology team. This included the National Institute of Health Stroke Score (NIHSS) at arrival (23). Two neurologists (MD, GC) used the modified Trial of Org 10172 in Acute Stroke Treatment (TOAST) classification (24, 25) to retrospectively categorize the stroke etiology based on the patient record. We also identified patients with cardioembolic stroke for whom a previously undiagnosed atrial fibrillation was discovered during their hospitalization. Data concerning functional independence at three months was collected by the Franche-Comté Stroke registry using the modified Rankin Scale (mRS) (26, 27).

### Time Data

The SO time was recorded prospectively by the on-call neurology team. When the exact time was uncertain (e.g., wake-up stroke or daytime-unwitnessed stroke), the team recorded the time at which the patient had last been seen well. We defined patients with unknown SO time as those who were last seen well more than 15 min prior. The other patients were considered to have a known onset time. We obtained the time of the first MRI sequence from the Besançon University Hospital image archiving and transfer system. The SO–imaging time was calculated in minutes.

### Radiological Data

We performed a retrospective analysis of the MRI data obtained in the acute phase. The exact MRI sequences and protocols used were at the discretion of the radiology teams. Every patient included had undergone a T2 standard gradient echo. We defined the presence of SVS as a hypointense signal on the T2^*^ images at the site of the occluded artery, for which the signal exceeded the diameter of the contralateral artery (28). The presence or absence of SVS was established visually by a junior neurologist (MD) and a senior interventional neuroradiologist (GC) as a single reading. For images that were difficult to classify, a consensus decision was made by the two readers. Doubtful cases were classified as absence of SVS. The site of the occlusion was defined using either the 3D-TOF sequence of the MRI, or the diagnostic angiography from the mechanical thrombectomy procedure. We calculated the infarct volume manually by drawing an outline on the diffusion sequence images. We also used the software Carestream (Carestream Health, Rochester, USA) to perform a semi-automatic calculation. The presence of a symptomatic hemorrhagic transformation on the CT scan at 24 h following the mechanical thrombectomy procedure was defined as an intraparenchymal hemorrhage with a volume 30% greater than the ischemic lesion, associated with an increase of 4 points on the NIHSS score, in accordance with the Heidelberg classification (29). A malignant infarction was defined retrospectively as a large lesion observed at the CT scan 24 h after the mechanical thrombectomy procedure, associated with cerebral herniation and clinical deterioration.

### Data Related to Mechanical Thrombectomy

The use of a stent retriever during the mechanical thrombectomy procedure was recorded either prospectively by the on-call neurology team, or retrospectively using the operative report. We used the Modified Treatment in Cerebral Ischemia score (mTICI) (30) to evaluate the level of brain tissue reperfusion following the procedure. The mTICI scores were reviewed by a junior neurologist (MD) and a senior interventional neuroradiologist (GC).

### Histopathological Data

Thrombi retrieved during the mechanical thrombectomy procedure were sent to the Besançon University Hospital pathology laboratory for analysis. Analysis included calculation of the proportion of red blood cells, white blood cells and fibrin, reported in percentages, measured by visual quantification by several pathologists as part of routine care. Histopathological analysis of retrieved thrombi had only been performed since August 2018.

### Statistics

To study the relationship between SVS and the SO–imaging time, we performed univariate and multivariate analyses of the whole study population, then of the subgroup with a known symptom onset time (KSO). We compared the patients with an SVS (SVS+), to the patients without (SVS–). The quantitative variables are reported as means (standard deviation) or as medians, and were analyzed using Welch's *t*-test. The qualitative variables are reported as a number (percentage) and were analyzed using the χ^2^ test or Fisher's exact test depending on the sample size. The variables with significant results in the univariate analysis, and those that seemed relevant according to the literature were then used in the multivariate analysis. We used logistical regression to perform the multivariate analysis. Statistical tests were performed using the software R. A *p*-value lower than 0.05 was considered significant.

## Results

### Population

**Figure 1:**
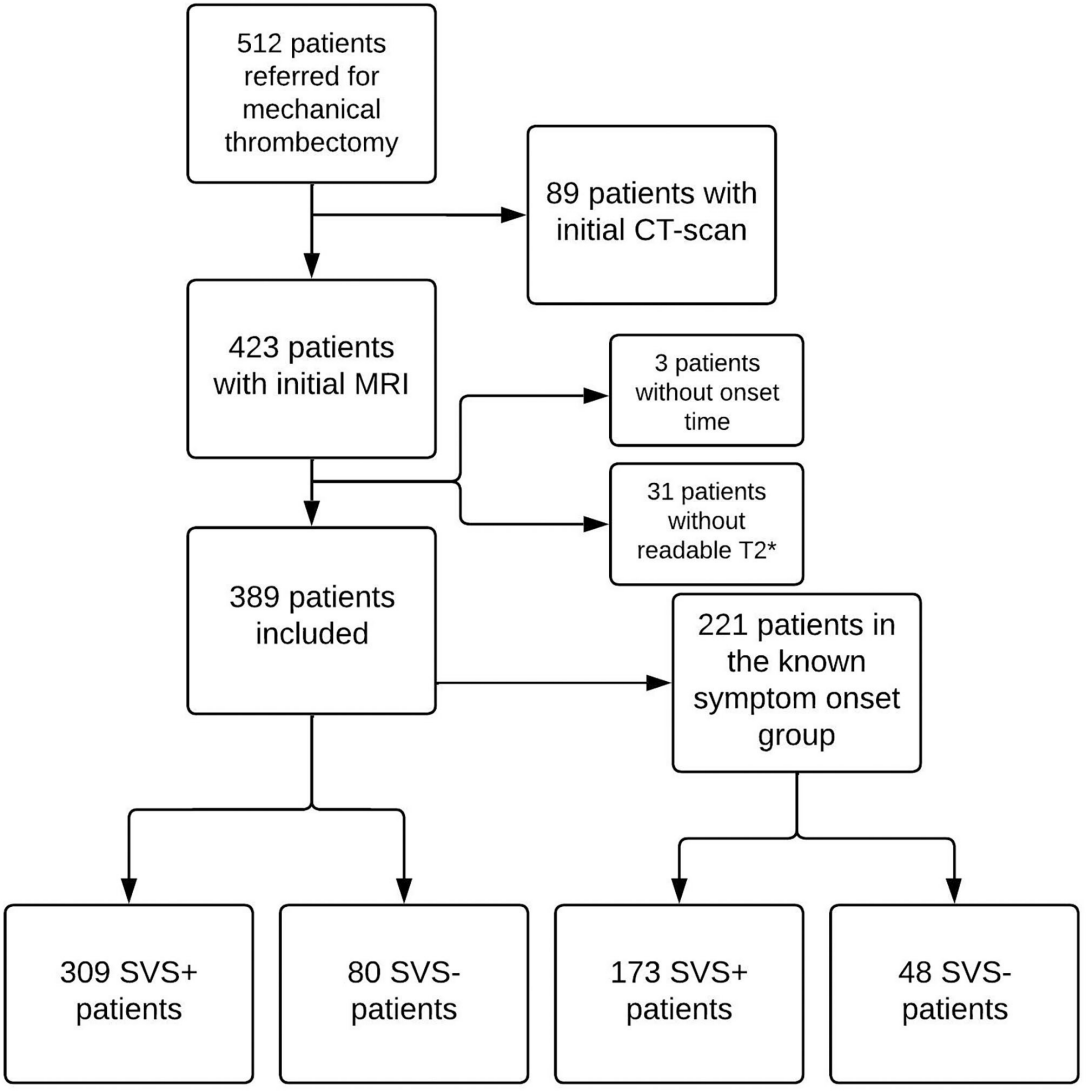
Study flowchart. CT, computed tomography; MRI, Magnetic Resonance Imaging; SVS, susceptibility vessel sign.

Between 1st of January 2015 and 31st of December 2020, 512 patients were referred for mechanical thrombectomy at Besançon University Hospital. Of these, 423 underwent an initial brain MRI. We excluded 34 of these patients, 31 due to lack of readable T2^*^ imaging and 3 with no SO time recorded in the patient file. We therefore included a total of 389 patients. Figure 1 shows the flowchart of the study.

### Population Characteristics

The demographic, radiological and follow-up data of the whole cohort is available in Supplementary Material. Of the 389 patients included in the study, 309 presented an SVS (79.4%). There was no significant difference between the SVS+ and SVS– groups in terms of SO–imaging time. SVS+ patients were significantly older than SVS– patients (*p* = 0.023). SVS was associated with stroke etiology (*p* = 0.01). For SVS+ patients, stroke etiology was more often cardioembolic than SVS–, whereas for SVS– patients, stroke was more often due to other determined etiology than SVS+. The SVS– patients were more often already taking anticoagulant medication (*p* = 0.018).

### Multivariate Analysis of the Whole Study Population

The results of the multivariate analysis in the whole cohort is available in Supplementary Material. The analysis included the age, anticoagulant treatment at admission, carotid terminus occlusion, and stroke etiology. SVS+ patients were older than SVS– patients (*p* = 0.023; OR 0.989; 95%CI [0.964; 0.997]), and presented with a carotid terminus occlusion significantly more frequently than SVS– patients (*p* = 0.042; OR 0.432; 95%CI [0.180; 0.922]). The SVS– patients were more often already taking an anticoagulant treatment than the SVS+ patients (*p* < 0.01; OR 2.84; 95%CI [1.48; 5.45]).

### Analysis of the Subgroup With Exact Known Symptom Onset Time (KSO)

**Figure 2:**
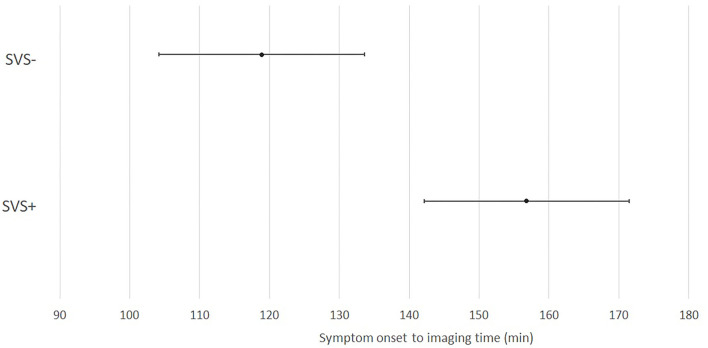
Repartition of susceptibility vessel sign over time from symptom onset to imaging in the KSO subgroup. SVS, susceptibility vessel sign; min, minutes.

The demographic, radiological and follow-up data of the KSO subgroup is presented in Table 1. This subgroup included 221 patients (56.9% of the whole study population). Of these, 173 presented an SVS (78.3%). SVS– patients had a significantly shorter mean SO–imaging time than SVS+ patients (*p* < 0.001). A graph showing mean SO-imaging time and 95% confidence interval depending on SVS in this subgroup is presented in Figure 2. SVS was associated with stroke etiology (*p* < 0.01). For SVS– patients, stroke etiology was more often other determined etiology than SVS+ patients. Among other determined etiology in this subgroup, neoplasia was the most common (5 patients), followed by cervical artery dissection (3 patients). We found no significant differences for the other variables analyzed.

**Table 1:** Characteristics of the subgroup with exact known symptom onset time.

	**SVS+**	**SVS–**	**Total**	***p***
Number (%)	173 (78.3)	48 (21.7)	221	
Age, mean (sd)	70.4 (15.9)	64.8 (17.6)	69.2 (16.4)	0.0504
Female, *N* (%)	95 (54.9)	25 (52.1)	120 (54.3)	0.73
Antiplatelet at admission, *N* (%)	46 (26.6)	11 (22.9)	57 (25.8)	0.61
Anticoagulant at admission, *N* (%)	26 (15.0)	11 (22.9)	37 (16.7)	0.2
Anticoagulant + antiplatelet at admission, *N* (%)	5 (2.9)	2 (4.2)	7 (3.2)	0.65
NIHSS at admission, med	17	14	17	0.38
Ischemic volume, mL, mean (sd)	53.4 (71.5)	37.4 (52.9)	49.9 (68.1)	0.091
**TOAST Classification** ***N*** **(%)**
Cardioembolic	96 (55.5)	20 (41.7)	116 (52.5)	**<0.01**
of which discovery of atrial fibrillation	60 (34.7)	9 (18.8)	69 (31.2)	–
Definite large-artery atherosclerosis	15 (8.7)	4 (8.3)	19 (8.6)	–
Possible large-artery atherosclerosis	18 (10.4)	2 (4.2)	20 (9.0)	–
Stroke of other determined etiology	4 (2.3)	7 (14.6)	11 (5.0)	–
of which cervical artery dissection	2 (1.2)	1 (2.1)	3 (1.4)	–
Stroke of undetermined etiology	40 (23.1)	15 (31.3)	55 (24.9)	–
**Thrombus location** ***N*** **(%)**
M1	111 (64.1)	31 (64.6)	142 (64.3)	0.061
M2	23(13.3)	12 (25.0)	35 (15.8)	–
Vertebrobasilar	12 (6.9)	0 (0)	12 (5.4)	–
Carotid terminus	27 (15.6)	5 (10.4)	32 (14.5)	–
Tandem	22 (12.7)	4 (8.3)	26 (11.8)	0.4
**Thrombectomy**, ***N*** **(%)**
Stent Retriever	44/152 (29.0)	12/43 (27.9)	56/195 (28.7)	0.89
TICI 2b−3 at end of procedure	136/169 (80.5)	36/43 (83.7)	172/212 (81.1)	0.63
**Follow-up**, ***N*** **(%)**
Malignant CI	19/170 (11.2)	2/47 (4.3)	21/217 (9.7)	0.26
sICH at 24 h	9/171 (5.3)	1/48 (2.1)	10/219 (4.6)	0.69
mRS 0–2 at 3 months	61/147 (41.5)	13/35 (37.1)	74/182 (40.7)	0.64
**Time**
Time SO–imaging, min, mean (sd)	157 (98)	118 (52)	148 (92)	**<0.001**
Time SO–imaging, min, med, *N*	138/173	114/48	130/221	

*SVS, susceptibility vessel sign; NIHSS, National Institute of Health Stroke Score; TOAST, Trial of Org 10172 in Acute Stroke Treatment; mTICI, Modified Treatment in Cerebral Ischemia score; sICH, Symptomatic intracranial hemorrhage; mRS, modified Rankin Scale; SO, symptom onset. Bold values are significant p-values (<0.05)*.

### Multivariate Analysis of the KSO Subgroup

The results of the multivariate analysis of the KSO subgroup are presented in Table 2. The analysis included the age, and stroke etiology. SVS– patients had a significantly shorter mean SO–imaging time than SVS+ patients (*p* = 0.011; OR 0.911; 95%CI [0.844; 0.972]).

**Table 2:** Multivariate analysis of the subgroup with known symptom onset time.

	**Odds Ratio**	***p***
Symptom onset—imaging time	0.911 [0.844; 0.972]	**0.011**
Age	0.988 [0.967; 1.01]	0.24
TOAST Cardioembolic vs. others	1.55 [0.774; 3.12]	0.22

*TOAST, Trial of Org 10172 in Acute Stroke Treatment. Bold values are significant p-values (<0.05)*.

### Histopathological Analysis of Thrombi

The results of the histopathological analyses of the thrombi are reported in Table 3. From August 2018, we recovered thrombi from 90 patients and sent them for analysis. An SVS was present in 71 of these patients (78.9%). The thrombi from SVS+ patients contained a greater proportion of red blood cells (*p* < 0.001), whereas the thrombi from SVS– patients contained a greater proportion of fibrin (*p* < 0.01). There was no significant difference between the two groups in terms of the proportion of white blood cells. There was no correlation between red blood cells quantity and SO-imaging time (p 0.34) on those 90 patients.

**Table 3:** Histopathological analysis of thrombi.

	**SVS+**	**SVS–**	**Total**	***p***
Number (%)	71 (78.9)	19 (21.1)	90	
% red blood cells, mean (sd)	56.7 (22.7)	28.9 (29.5)	50.8 (26.7)	**<0.001**
% white blood cells, mean (sd)	12.8 (13.6)	16.5 (20.2)	13.6 (15.2)	0.92
% Fibrin, mean (sd)	30.9 (20.2)	54.1 (31.9)	35.8 (24.9)	**<0.01**

*SVS, susceptibility vessel sign; sd, standard deviation. Bold values are significant p-values (<0.05)*.

## Discussion

### Main Result

In our analysis of the total study population, we found no statistically significant relationship between the absence of an SVS and a shorter SO–imaging time. However, we did find a significant relationship between these two factors in the KSO subgroup in both the univariate and multivariate analyses (Tables 1, 2).

To our knowledge, this is the first study of SVS to analyze the relationship between SVS and time in correlation with clot histopathology. This is also the first study of SVS to assess the link between SVS and SO–imaging time in a subgroup of KSO patients. Previous studies of cohorts with a mixture of patients with known and unknown symptom onset times have reported inconsistent results (2, 6, 8, 17, 19, 31). We could hypothesize that the absence of the relationship between SVS and SO–imaging time in the previous studies (2, 6, 8, 19, 31) was due to an insufficient sample size or the inaccuracy of the symptom onset time reported by the patients or their families.

Our study could indicate that in patients with unknown symptom onset time, the absence of an SVS could support the hypothesis of a more recent thrombus. However, it seems essential to study the different factors that influence this radiological biomarker before it is used to make clinical decisions. In our study, there was no significant difference between the SVS+ patients and SVS– patients in terms of the SO–imaging time in the whole study population, but the means and medians show the same general trend as the KSO subgroup. The lack of statistical significance is likely due to wake-up strokes, for which the SO time is taken as the time the patient went to bed, thus significantly extending the SO-imaging time.

Moreover, our study confirmed that the presence of an SVS is associated with a thrombus containing mainly red blood cells, whereas its absence is associated with a thrombus with a high proportion of fibrin (Table 3), as reported in the literature (11–13, 32, 33). The composition of an intracranial thrombus varies over time, and we could therefore hypothesize that the likelihood of the presence of an SVS in the acute phase increases over time in connection with an increase in deoxyhemoglobin in its core. Therefore, in our opinion, uncertainty related to SO time reported by patients or their relatives should be considered in upcoming studies on SVS, as results vary when SO time is certain. However, we found no correlation between SO-imaging time and proportion of red blood cells within thrombi. It is questionable whether SVS truly reflects the proportion of red blood cells within the clot, or only the deoxyhemoglobin in its core. Yet, recent studies suggest that composition of intracranial thrombi are highly heterogenous (34), so it is questionable whether SVS could be a better marker of thrombus composition than routine care histopathology.

Several studies discuss the correlation between SO-imaging time and density of the hyperdense middle cerebral artery sign on CT scan (35–37). One of those found a time-dependent loss of density in the M1 segment in the first 5 h (36). However, the relationship between SVS and the hyperdense artery sign is questionable (14, 38, 39), and it is difficult to assess in a large group of patients, because it requires both a CT scan and an MRI in the acute phase.

### Other Parameters Studied and Comparison With Literature

In our whole cohort, 79.4% of patients presented with an SVS, which is in line with data reported in recent studies (71 to 86% of patients) (2, 7, 17, 40–42).

Of the 389 patients in our cohort, 221 (56.8%) were included in the KSO subgroup, which may seem relatively low compared to the literature (43). To our knowledge, stroke with unknown onset time was not defined in previous studies, aside from wake-up strokes. We purposefully chose a short time period (15 min) to evaluate the impact of SO–imagery time on SVS as precisely as possible, and this short time could explain the low number of patients in the KSO subgroup.

SVS+ patients were older than SVS– patients, as found in a recent study (17). In SVS+ patients, the most common stroke etiology was cardioembolic, which is also in accordance with the literature (1–4). This result was not confirmed in the multivariate analysis. This may corresponds to the increased prevalence of atrial fibrillation with age (44, 45).

SVS– patients were more often undergoing anticoagulant treatment on arrival, most commonly due to atrial fibrillation. We can hypothesize that patients already taking anticoagulant treatment present with “young” thrombi, which contain little deoxyhemoglobin and little red blood cells in their core. In patients with atrial fibrillation, cardiac embolism is caused by stagnation of the blood in the left atrium (46, 47). Curative anticoagulation could therefore prevent the accumulation and stagnation of the blood, and the formation of a cardiac thrombus. It is therefore possible that in these patients, the intracranial thrombi are more recent and contain less deoxyhemoglobin. Therefore, it is questionable whether atrial fibrillation or curative anticoagulation influences SVS the most.

In SVS– patients, stroke etiology was more frequently “other determined etiology” than in SVS+ patients. We could hypothesize that this result is related to the prevalence of active neoplasia, which is included within the “other determined etiology” category of the TOAST classification, as neoplasia is known to lead to thrombi that are high in fibrin and platelets (48).

The understanding of clot formation has improved in recent years with reports of analyses of retrieved clots. Clot composition may be predictive of stroke etiology, but this is debated in the literature, with controversial findings among the studies (33, 49–51). The composition of the clot could also affect the outcome of thrombolysis or mechanical thrombectomy (52).

SVS+ patients more frequently presented with carotid terminus occlusion than SVS– patients. Because the slice thickness is set at 3 mm, it is possible that the middle cerebral artery thrombi were detected less often on the axial gradient echo images used for this cohort than carotid artery thrombi (53).

Our study did not find any statistically significant relationship between SVS and recanalization mTICI following the mechanical thrombectomy procedure. The interest of SVS as a predictor of complete recanalization has been studied several times in recent years, but the results of the different studies vary greatly (1, 3, 7, 19, 54). We found no association between SVS and functional independence at 3 months, which is also debated in the literature (1, 5, 55).

### Limits

Our study has some limitations. This study was conducted retrospectively. We excluded patients who underwent an MRI without an interpretable T2^*^ sequence, and those whose time of SO was not recorded in the medical file, which could represent a selection bias. The brain MRI scans were performed using different scanners, with different protocols and different parameters, which could lead to different SVS detection sensitivities. As presence or absence of SVS was assessed as a single reading, we can't provide an inter-reader agreement. However, a recent study shows that the rating of SVS is reproductible (17). It is possible that the SO times we obtained were not completely reliable, as the majority of these were reported by the patients or the people accompanying them, and may therefore not be completely accurate. We only included patients who had an indication for mechanical thrombectomy, and so we therefore did not include patients with an SO–imaging time >24 h, or who had a distal occlusion. Additionally, in the KSO subgroup, only 6 patients had a SO–imaging time >6 h, limiting the interest of those values. However, this is the first study on SVS to assess the subgroup of KSO patients, which allowed us to gain a clearer picture of the relationship between the thrombus formation time and the SVS.

## Conclusion

In the KSO subgroup, the absence of an SVS in the acute phase of proximal occlusion ischemic stroke was associated with a shorter SO–imaging time. Therefore, uncertainty related to SO time reported by patients or their relatives should be considered in future studies on SVS. SVS is a complex radiological sign, that is also impacted by curative anticoagulation or stroke etiology, and further investigations are needed before using SVS in clinical practice.

## Data Availability Statement

The raw data supporting the conclusions of this article will be made available by the authors, without undue reservation.

## Ethics Statement

Ethical review and approval was not required for the study on human participants in accordance with the local legislation and institutional requirements. Written informed consent for participation was not required for this study in accordance with the national legislation and the institutional requirements.

## Author Contributions

MD and GC conceived this study, contributed to data collection and analysis, and wrote the manuscript in consultation with AB, LB, TM, and FV. All authors have read and approved the manuscript.

## Conflict of Interest

The authors declare that the research was conducted in the absence of any commercial or financial relationships that could be construed as a potential conflict of interest.

## Publisher's Note

All claims expressed in this article are solely those of the authors and do not necessarily represent those of their affiliated organizations, or those of the publisher, the editors and the reviewers. Any product that may be evaluated in this article, or claim that may be made by its manufacturer, is not guaranteed or endorsed by the publisher.

## Abbreviations

KSO: Known Symptom Onset
SO: Symptom Onset
SVS: Susceptibility Vessel Sign.
