# Supplementary material for: Factors That Influence Susceptibility Vessel Sign in Patients With Acute Stroke Referred for Mechanical Thrombectomy
Source: Front Neurol. 2022 May 11;13:893060. doi: 10.3389/fneur.2022.893060 (PMC9130602; doi:10.3389/fneur.2022.893060)
Supplement: Supplementary file 1 [file Table_1.DOCX]

|  | **SVS+** | **SVS-** | **Total** | ***p*** |
| --- | --- | --- | --- | --- |
| Number (%) | 309 (79.4) | 80 (20.6) | 389 |  |
| Age, mean (sd) | 71.8 (14.5) | 66.8 (17.6) | 70.7 (15.3) | **0.023** |
| Female, N (%) | 164 (53.1) | 47 (58.8) | 211 (54.2) | 0.36 |
| Antiplatelet at admission, N (%) | 83/305 (27.2) | 17/78 (21.8) | 100/383 (26.1) | 0.33 |
| Anticoagulant at admission, N (%) | 47/305 (15.4) | 21/78 (26.9) | 68/383 (17.8) | **0.018** |
| Anticoagulant + antiplatelet at admission, N (%) | 9/305 (3.0) | 4/78 (5.1) | 13/383 (3.4) | 0.31 |
| NIHSS at admission, med | 17 | 16 | 17 | 0.33 |
| Ischemic volume, mL, mean (sd) | 50.7 (69.1) | 37.7 (48.9) | 48.0 (65.6) | 0.055 |
| **TOAST Classification N (%)** |  |  |  |  |
| Cardioembolic | 174 (56.3) | 35 (43.8) | 209 (53.7) | **0.01** |
| of which discovery of atrial fibrillation | 104 (33.7) | 11 (13.8) | 115 (29.6) | **-** |
| Definite large-artery atherosclerosis | 28 (9.1) | 6 (7.5) | 34 (8.7) | - |
| Possible large-artery atherosclerosis | 25 (8.1) | 4 (5.0) | 29 (7.5) | - |
| Stroke of other determined etiology | 12 (3.9) | 11 (13.8) | 23 (5.9) | **-** |
| of which cervical artery dissection | 8 (2.6) | 3 (3.8) | 11 (2.8) | - |
| Stroke of undetermined etiology | 70 (22.7) | 24 (30.0) | 94 (24.2) | - |
| **Thrombus location N (%)** |  |  |  |  |
| M1 | 185 (59.9) | 53 (66.3) | 238 (61.2) | 0.06 |
| M2 | 44 (14.2) | 17 (21.2) | 61 (15.7) | - |
| Vertebrobasilar | 19 (6.1) | 2 (2.5) | 21 (5.4) | - |
| Carotid terminus | 61 (19.7) | 8 (10.0) | 69 (17.7) | **-** |
| Tandem | 44 (14.2) | 6 (7.5) | 50 (12.9) | 0.11 |
| **Thrombectomy, N (%)** |  |  |  |  |
| Stent Retriever | 100/276 (36.2) | 20/69 (29.0) | 120/345 (34.8) | 0.26 |
| TICI 2b–3 at end of procedure | 241/301 (80.1) | 63/74 (85.1) | 304/375 (81.1) | 0.32 |
| **Follow-up, N (%)** |  |  |  |  |
| Malignant CI | 35/301 (11.6) | 4/78 (5.1) | 39/379 (10.3) | 0.092 |
| sICH at 24 hours | 16/304 (5.3) | 3/79 (3.8) | 19/383 (5.0) | 0.77 |
| mRS 0–2 at 3 months | 97/259 (37.5) | 23/63 (36.5) | 120/322 (37.3) | 0.89 |
| **Time** |  |  |  |  |
| Time SO–imaging, min, mean (sd) | 252 (220) | 211 (204) | 243 (217) | 0.12 |
| Time SO–imaging, min, med, N | 169/309 | 131/80 | 162/389 |  |
|  |  |  |  |  |

**Supplementary Material
Table 4 – Whole cohort characteristics**Legend: SVS: susceptibility vessel sign ; NIHSS: National Institute of Health Stroke Score ; TOAST: Trial of Org 10172 in Acute Stroke Treatment ; mTICI: Modified Treatment in Cerebral Ischemia score ; sICH: Symptomatic intracranial hemorrhage ; mRS: modified Rankin Scale ; SO: symptom onset

**Table 5 - Multivariate analysis of the whole cohort**Legend: TOAST: Trial of Org 10172 in Acute Stroke Treatment

|  | **Odds Ratio** | ***p*** |
| --- | --- | --- |
| Symptom onset – imaging time | 0.920 [0.800; 1.04] | 0.21 |
| Age | 0.989 [0.964 ; 0.997] | **0.023** |
| Pre-existing anticoagulant treatment | 2.84 [1.48 ; 5.45] | **<0.01** |
| Carotid terminus occlusion | 0.432 [0.180 ; 0.922] | **0.042** |
| TOAST Cardioembolic versus others | 1.76 [0.993 ; 3.14] | 0.054 |
